# Potentially Inappropriate Prescribing among Elderly Outpatients: Evaluation of Temporal Trends 2012–2018 in Piedmont, Italy

**DOI:** 10.3390/ijerph19063612

**Published:** 2022-03-18

**Authors:** Federica Galimberti, Manuela Casula, Lorenza Scotti, Elena Olmastroni, Daniela Ferrante, Andrealuna Ucciero, Elena Tragni, Alberico Luigi Catapano, Francesco Barone-Adesi

**Affiliations:** 1 IRCCS MultiMedica, Sesto S. Giovanni, 20099 Milan, Italy; federica.galimberti@multimedica.it (F.G.); alberico.catapano@unimi.it (A.L.C.); 2 Epidemiology and Preventive Pharmacology Service (SEFAP), Department of Pharmacological and Biomolecular Sciences, University of Milan, 20133 Milan, Italy; elena.olmastroni@unimi.it (E.O.); elena.tragni@unimi.it (E.T.); 3 Department of Translational Medicine, University of Eastern Piedmont, 28100 Novara, Italy; lorenza.scotti@uniupo.it (L.S.); daniela.ferrante@med.uniupo.it (D.F.); andrealuna.ucciero@uniupo.it (A.U.); francesco.baroneadesi@uniupo.it (F.B.-A.)

**Keywords:** potentially inappropriate prescribing, elderly, primary care, healthcare databases

## Abstract

Pharmacological intervention is one of the cornerstones in the treatment and prevention of disease in modern healthcare. However, a large number of drugs are often prescribed and used inappropriately, especially in elderly patients. We aimed at investigating the annual prevalence of potentially inappropriate prescriptions (PIPs) among older outpatients using administrative healthcare databases of the Piedmont Region (Italy) over a seven-year period (2012–2018). We included all Piedmont outpatients aged 65 years or older with at least one drug prescription per year. Polypharmacy and the prevalence of PIPs according to the ERD list explicit tool were measured on an annual basis. A range between 976,398 (in 2012) and 1,066,389 (in 2018) elderly were evaluated. Among them, the number of subjects with at least one PIP decreased from 418,537 in 2012 to 339,764 in 2018; the prevalence significantly reduced by ~25% over the study period. The stratified analyses by age groups and sex also confirmed the downward trend and identified several differences in the most prevalent inappropriately prescribed drugs. Overall, despite a reduction in PIP prevalence, one out of three older outpatients was still exposed to inappropriateness, highlighting the extensive need for intervention to improve prescribing.

## 1. Introduction

Medicines play a crucial role in helping people all over the world to live longer, healthier, and more productive lives [1]. However, their inappropriate prescription and use, affecting one in three older persons in primary care [2], has a significant negative clinical and economic impact, and it has become an important public health issue worldwide [3,4]. Older people are particularly at risk of the adverse consequences of inappropriate pharmacological treatments. Indeed, the physiological and pathological changes with aging could modify pharmacokinetics and pharmacodynamics. This is even more critical when several drugs are used to treat multiple pathological conditions and when several healthcare professionals are involved in the patient’s care without sharing the therapeutic decision-making process [5]. Therefore, prescribing in older people, in the context of multimorbidity, is complex, as adverse effects relating to medications in the elderly are more common than in younger individuals [6,7,8].

Potentially inappropriate prescribing has been defined as the prescription of a medication without a clinical indication or the prescription of an indicated medication in conditions where the risks outweigh the benefits or where a safer alternative exists [9]. It has been associated with the clinical failure of disease management, a significant increase in the risk of adverse drug events, functional decline, worsening in health-related quality of life, and a higher risk of hospitalization and mortality [4,10]. The epidemiological and clinical impact of inappropriate drug prescribing warrants the design and implementation of tools to measure the number of exposed patients and to identify areas deserving prompt intervention.

In recent years, many strategies and tools have been developed to assess the inappropriate prescription of medications with adverse health impacts on the patient [11]. Among explicit process measures or criteria [9,12], which verify whether the prescriptions comply with accepted standards and are suited to be applied systematically on a large number of patients with little or no clinical judgment, the American Geriatrics Society (AGS) Beers Criteria [13] or the Screening Tool of Older Persons’ Potentially Inappropriate Prescriptions (STOPP) and the Screening Tool to Alert Doctors to the Right Treatment (START) [14] have been used extensively in research, both to assess the prevalence of PIPs [15,16] and their association with patient and economic outcomes [17] and as an outcome measure for interventions to improve prescribing practices [18]. Explicit criteria are generally used as rigid standards and each country has specific guidelines, standards, and approved medications, which usually makes a country-specific adaption necessary. On the other hand, they are an easy-to-apply and low-cost tool, which allows one to carry out robust and reproducible measurements over time, thereby monitoring trends in the prevalence of prescriptive inappropriateness [12]. They allow the quantitative assessment of the inappropriate prescription phenomenon and the identification of the most involved therapeutic classes. This is of paramount importance to inform health decision-makers in designing appropriate and effective interventions. 

In this analysis, our objective was to apply an Italian-specific explicit tool to evaluate the annual prevalence of potentially inappropriate prescribing in the elderly population of a large Italian region over a seven-year period (2012–2018).

## 2. Materials and Methods

### 2.1. Data Sources

Italy has a universal coverage National Health System (NHS) organized at three levels: national, regional (21 regions), and local (on average 10 Local Health Authorities—LHA—per region). Italian healthcare administrative data contains a wealth of information on healthcare service utilization, including pharmacy claims from community and hospital pharmacies. They have a high level of coverage because data collection on a regional basis is mandated by national law across the whole country.

This study was conducted using the administrative health databases of Piedmont, an Italian region with a population of 4,400,000 inhabitants approximately (corresponding to 7.5% of the national Italian population). We used the following databases: (i) the inhabitants registry, containing demographic information such as gender, birth date, death date, and other relevant demographic information, and (ii) the outpatient prescription registry, including the information of all outpatient drug prescriptions reimbursable by the NHS, such as dispensation date and ATC codes.

### 2.2. Study Design and Setting

In our analysis, we evaluated data related to the period 2012–2018. The study population comprised all Piedmont residents aged ≥65 years. Subjects had to receive at least one medication reimbursed by the NHS during the study duration to be included in the study. Subjects were also excluded in case of drop-out during the year, e.g., due to death or migration.

### 2.3. Evaluation of PIP

PIPs among the elderly were evaluated using the ERD list [19]. Briefly, this list was drawn up by merging the 2015 Beers criteria [20], the STOPP and START criteria [21], and the EU-(7)-PIM list [22], and then adapting them to the Italian setting by selecting only the drugs available on the Italian market and reimbursed by the Italian NHS. The list includes only drugs for which there was a clear recommendation to always be avoided in elderly patients, as other criteria (considering drugs that should be used with caution or avoided in certain patients with certain diseases or conditions) would require clinical information not available in administrative databases [23]. In addition, drugs not reimbursed by the 2016 Italian National Formulary were not included in the list, as they are not traced in administrative pharmacy databases.

The full list of PIPs considered in the current study is reported in Table A1.

### 2.4. Definition of Polypharmacy

Polypharmacy is defined as a two-level indicator: dispensing of 5–9 or greater than or equal to 10 different drugs (ATC code—fifth level) within the same quarter of each year. The number of medicines dispensed in each quarter was calculated and the highest number of drugs dispensed in a single quarter was used to define polypharmacy over the 1-year period [24].

### 2.5. Statistical Analysis

Descriptive statistics were used to summarize the main characteristics of older patients with at least one PIP. Continuous variables were reported as mean and corresponding standard deviation, or median and first and third quartile if not normally distributed. Categorical variables were reported as absolute frequencies and percentages.

For each PIP, we calculated: (i) the annual prevalence of exposure among patients (ratio between the number of subjects exposed to the PIP and the number of subjects with at least one prescription during the year) and (ii) the annual prevalence of exposure among patients with PIPs (ratio between the number of subjects exposed to the PIP and the number of subjects with at last one PIP). Every year, the prevalence was calculated on the whole sample and stratified by age classes (65–74, 75–84, 85–94, ≥95 years) and sex. The 5 most common PIPs for each year were also reported. 

To further analyse the differences in the proportion of PIPs between sexes and age classes and to evaluate the presence of a statistically significant time trend, subjects included in the study during 2012 were selected and followed longitudinally until 2018. A repeated measure logistic regression model was fitted to estimate the odds ratios (ORs) and corresponding 95% confidence intervals (95%CI) for the relationship between age, sex, and calendar year and the probability of being exposed to at least one PIP accounting for the correlation of the observations within subjects. 

Data analysis was performed using SAS (Statistical Analysis System) software version 9.4 (SAS. Institute, Inc. Cary, NC, USA), and two-tailed *p* <0.05 was considered for statistical significance in all analyses.

### 2.6. Ethical Consideration

All procedures conducted in this study were in accordance with the ethical standards of the institutional and/or national research committee and with the 1964 Helsinki Declaration and its later amendments or comparable ethical standards. The study protocol was approved by the local ethical committee of the Hospital “Maggiore della Carità”, Novara (CE 144/19). The study was conducted using data routinely collected in the aforementioned regional administrative healthcare databases, in which authors had access to anonymized data only, hence informed consent was not required.

## 3. Results

In Piedmont, patients ≥65 years receiving at least one prescribed drug ranged from 976,398 to 1,066,389 in the 2012–2018 period. 

**Figure 1:**
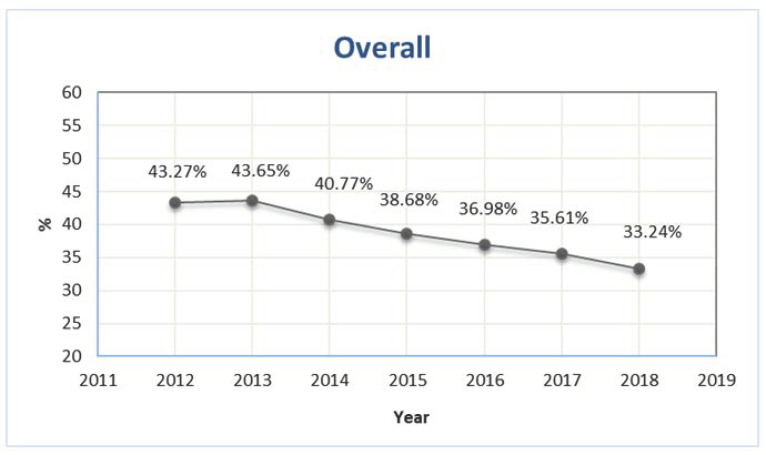
Prevalence of outpatients older than 65 years exposed to at least one potentially inappropriate prescription in Piedmont in the period 2012–2018.

The number of older patients exposed to at least one PIP was 418,537 and 429,670 in 2012 and 2013, respectively, corresponding to 43.3% and 43.7% of the total number of older outpatients with at least one prescription (Figure 1). During the study period, the prevalence showed a sharp decrease, reaching 33.2% in 2018 (Figure 1).

Among these subjects with PIPs, the proportion of males was quite stable (range 38.6-39.0%) across years. The mean age showed a slight increase, from 75.7 in 2012 to 76.1 in 2018, as well as the proportion of subjects older than 80 years old, from 26.1% in 2012 to 28.2% in 2018 (Table 1). The median number of medications per patient was six, without relevant variation during the study period; the distribution according to polytherapy classes remained also stable (Table 1).

The prescription of proton pump inhibitors for at least 8 weeks was the most frequent PIP in 2012 and 2013, while the prescription of diclofenac was the most frequent PIP in the period 2014–2018, accounting for more than 20% of PIPs (Table 2). Ketoprofen was third in all the years evaluated, followed by tramadol (2012–2015) or sliding scale insulin (that is, an antidiabetic approach consisting of the correction of hyperglycemia through the frequent administration of short-acting insulin, dosed according to a patient’s blood glucose level; 2016–2018). At fifth in the ranking, there was the prescription of ticlopidine in 2012–2014 and paroxetine in 2017–2018 (Table 2).

**Figure 2:**
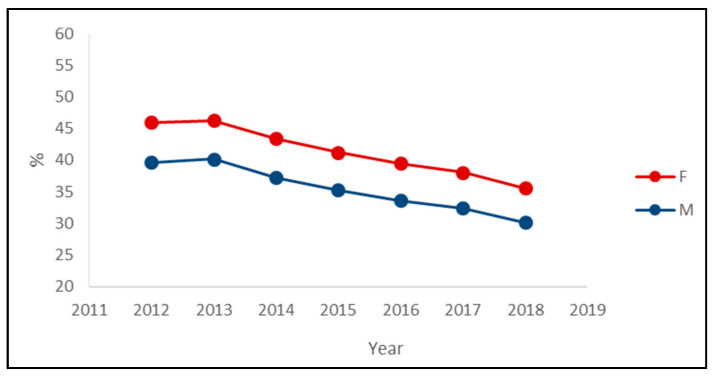
Prevalence of exposure to at least one potentially inappropriate prescription among the drugs’ users by the sex of outpatients older than 65 years in Piedmont in the period 2012–2018.

The decreasing trend in the prevalence of PIPs was observed in both sexes, with the prevalence among women being about six percentage points greater than the prevalence among men in each year (Figure 2). Regarding the most prevalent PIPs, the main differences between sexes were the presence of insulin in the male rank and the presence of paroxetine in the female rank (Table 3). 

**Figure 3:**
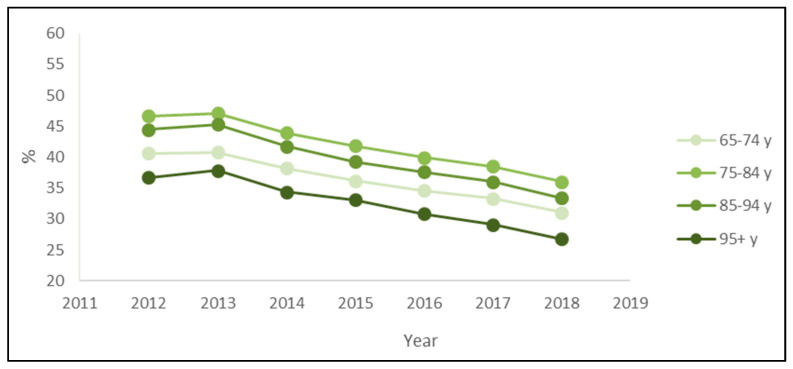
Prevalence of exposure to at least one potentially inappropriate prescription among the drugs’ users by the age classes of outpatients older than 65 years in Piedmont in the period 2012–2018.

The stratified analysis by age groups also confirmed the downward trend. Every year, the prevalence of PIPs was higher for patients 75–84 years old and lower for patients over 95 (Figure 3). Regarding the most prevalent PIPs, the main differences across age classes were the presence of ticlopidine only in the rank of over 95 patients and the presence of ketoprofen only in the rank of patients aged 65–74 or 75–84 years (Table 3).

Consistent with previous results, females have a probability 1.25 times higher than males to be exposed to at least one PIP. Regarding age, a statistically significant increase in the probability of PIP was observed for subjects aged 75–85 years (OR 1.144, 95%CI 1.138–1.150) and 85–95 years (OR 1.083, 95%CI 1.075–1.091) compared to those aged 65–75 years, while a decreased risk was observed for the oldest subjects (OR 0.886, 95%CI 0.867–0.905). Furthermore, the likelihood of being exposed to at least one PIP decreased by 6% every year (Table 4).

## 4. Discussion

Our analysis shows that 33.2% of older patients with at least one drug prescription received one PIP in 2018. Considering the whole Piedmont elderly population (about 1.1 million), this results in a proportion of 30.7%. This is in line with results from a recently published meta-analysis [4], showing that inappropriate prescribing affected one in three older persons in primary care (prevalence 33.3%, 95% CI 29.7–37.0%), based on 5 million participants from 27 countries. In particular, the pooled prevalence of the two Italian studies included was 32.0% (95% CI 23.7–41.0%). 

We also reported that prevalence was higher among women (vs men) and in the 75–84 and 85–94 age classes compared to 65–74 years. This evidence was confirmed by the stratified pooled analyses by Liew et al. [4], though the differences were not statistically significant, and the same pattern was described by Amos et al. in a study conducted in Central Italy [25]. 

Despite the still-high prevalence of PIPs, our analysis showed a clear reduction in PIP prevalence over 7 years, from 43.27% in 2012 to 33.24% in 2018 (23% decrease), more evident in subjects 85–94 years old (−25%) and 95 years old or more (−27%). As PIPs represent a matter of concern for their potential clinical consequences, this observed reduction in PIP prevalence might have led to a decrease in the incidence of associated events, such as hospitalizations and adverse drug reactions (ADRs). In 2019, a meta-analysis evaluating the association between PIPs—defined according to the Beers and STOPP criteria—and selected outcomes in the elderly population showed a statistically significant increased risk of hospitalizations and ADRs of 14% and 34%, respectively, compared to the absence of PIPs [4]. According to the prevalence of PIPs obtained in our study and the relative risk derived from the meta-analysis, the population attributable fraction of hospitalizations and ADRs could have been reduced from 6% to 4% and from 13% to 10%, respectively, during the 7-year period.

This trend of progressive improvement has been observed in other studies conducted in different countries. In Sweden, from 2006 to 2013, Hovstadius et al. [26] showed an improvement of specific appropriateness indicators; for example, the prescription of long-acting benzodiazepines decreased from 3.5% to 1.7% (−52.2%). Consistent with our results, the greatest reduction was seen in the oldest groups. More recently, in an evaluation among people aged 75 years and over between 2011 and 2019 in France [27], the prevalence of PIPs decreased from 49.6 to 39.6% over the study period, with a steeper decrease in the 75–84 age class. In a cross-sectional study conducted on middle-aged subjects in the setting of London primary care and using the PRescribing Optimally in Middle-aged People’s Treatments (PROMPT) criteria to define PIPs [28], the prevalence decreased from 20% in 2014 to 18% in 2019. Similarly, the point prevalence of the use of potentially inappropriate medications in the US [29], assessed by Beers criteria 2012, decreased from 37.6% in 2007 to 34.2% in 2012, with a statistically significant 2% decline per year (assuming a linear trend). Overall, the body of evidence seems to suggest a reduction in the epidemiological impact of PIPs on the elderly population starting from the early 2000s. Although in the analysed period there were no major national or European interventions aimed specifically at improving appropriate prescribing, it is possible that small local interventions have contributed to an increase in the awareness of health professionals regarding this issue. In addition, the growing use of computerized prescriptions has allowed the integration of digital prescription support tools aimed at automatically alerting prescribers when patients are at risk of interacting or inappropriate drugs. In some countries, this improvement could also be due to the spread of pharmaceutical care and medication review services [30], which in local experiences have been shown to improve prescriptive quality and clinical outcomes [31].

This trend is also observed for the prevalence of the most-reported inappropriate drugs: the prevalence of subjects exposed to diclofenac decreased from 10.7 to 9.1% (−15%), that of subjects exposed to proton pump inhibitors for more than 8 weeks from 8.8% to 5.8% (−34%), and that of subjects exposed to ketoprofen from 5.9 to 3.6% (−39%).

The temporal trends also show some variations of these drugs in terms of rank: overall, diclofenac was consistently the most reported, accounting for over a quarter of inappropriate prescriptions in 2018, followed by proton pump inhibitors (about 18%) and ketoprofen (about 11%). Other studies in the elderly population also confirmed a high prescription of NSAIDs and proton pump inhibitors [28,32]. NSAIDs are the most commonly prescribed drugs worldwide, as well as specifically in older patients, as they often suffer from musculoskeletal problems such as muscle strain, lower back pain, and knee osteoarthritis [33]. NSAIDs effectively alleviate the pain of such conditions, but they are also responsible for approximately 25% of all reported adverse drug reactions, and aging may substantially increase the risk of NSAID-induced reactions [34]. Age-related alterations in pharmacokinetics may influence the handling of NSAIDs in this age class. Their use is accompanied by a two- to five-fold risk of serious complications of peptic ulcer disease, as well as a broad range of renal side effects in the case of long-term exposure [35]. From this perspective, the lower prevalence of prescribing diclofenac and ketoprofen with increasing age seems to suggest a growing awareness of doctors. The same attention does not appear to be paid to proton pump inhibitors, which, perhaps thanks to a largely recognized reputation for effectiveness and good tolerability, remain widely used, even for long periods of time, by elderly and very elderly patients. Many studies have extensively described the issue of the inappropriate prescription of proton pump inhibitors, both for acute and long-term therapies [36]. In a large Italian cohort of hospitalized older patients from 2010 to 2016, 60% of the prescriptions of proton pump inhibitors at discharge were inappropriately prescribed [37]. In a cross-sectional study on community-dwelling older adults, 68% of the participants were found to have taken a proton pump inhibitor for a longer period than recommended by the national guidelines [38]. Over the years, there has been a growing concern over the potential adverse effects associated with long-term therapy with proton pump inhibitors, including hypergastrinemia, the development of pneumonia, dementia, as well as the risk of fractures, hypomagnesemia, *Clostridium difficile*-associated diarrhoea, vitamin B12 deficiency, acute interstitial nephritis, and cutaneous and systemic lupus erythematosus events [39]. However, physicians’ and patients’ awareness regarding this issue remains poor. In a survey on 120 elderly patients, the majority of the participants were not familiar with any reports linking long-term proton pump inhibitor use with adverse effects, reported no concerns related to their chronic use, and stated that they had not discussed the benefits and risks of this therapy with their primary care providers [40,41]. It is also to be acknowledged that older adults often report feeling uncomfortable discussing whether to stop proton pump inhibitors with their providers [42].

In our study, we also reported a trend towards a lower epidemiological impact of the inappropriate prescription of ticlopidine and tramadol, but an increasing prevalence of paroxetine and insulin prescriptions (each accounting for just under 3% of the cohort in 2018). The use of tramadol remains relevant in subjects over 85 years (2.4–3.2%). Tramadol is an effective weak opioid analgesic, and concerns about gastrointestinal bleeding and renal insufficiency risks associated with NSAIDs might favour tramadol as a safer alternative. However, the Beers criteria recommend that tramadol should be avoided or used with caution in older adults due to the risk of central nervous system (CNS) adverse effects. Moreover, the concomitant use of selective serotonin reuptake inhibitors (SSRIs), benzodiazepines, or first-generation antihistamines may increase serotonin syndrome risk [43]. An analysis of drug prescribing from 2000 to 2010 among community-dwelling elderly people aged 65–94 years in Lombardy, Italy, reported that analgesics (particularly opioids) showed the highest increase in use over time [44], and evidence showed that in some cases tramadol was prescribed in older patients, most of whom had significant co-morbidity and concomitant therapy, though not following expert pain management guidelines [45].

Paroxetine is widely used in the elderly, especially in older women, to treat depression, which is the most common mental health problem in this age group and is associated with a significant burden of illness that affects patients, their families, and communities, and takes an economic toll as well [46]. However, it has strong anticholinergic and sedative properties, which can lead to negative effects on cognition. Anticholinergic medications such as paroxetine are often considered potentially inappropriate for elderly patients with dementia and cognitive impairment [47]. The use of paroxetine and other SSRIs has substantially increased during the last decades [48], but studies show that patients often receive suboptimal treatment because of the concomitant use of anticholinergic drugs, excessively high or low daily dosages, short-duration therapy, or inadequate follow-up [49,50]. Confirming our results, a cross-sectional study in France showed that anti-depressive drugs represented 28.4% of the potentially inappropriate use of psychotropic drugs according to the 2003 Beers criteria list [51].

In the top five ranking of inappropriate drugs, insulin has been found in males, regardless of age class. It is important to remember that the effectiveness of sliding-scale insulin regimens has been questioned, and numerous diabetic best-practice treatment guidelines recommend its discontinuation [52]. One of the largest cohort studies to date found that 76% of general medical inpatients received sliding-scale insulin, with these regimens not only failing to control hyperglycaemia but also resulting in more episodes of hypoglycaemia and longer hospital stays [53].

The main strength of our study is the use of the ERD list, which was designed in the context of a large Italian study [19] on the basis of lists already published in the literature and extensively validated; the ERD list is specific to the Italian geographical setting and the type of data source. The administrative databases themselves are an element of strength, as they collect all the reimbursed drugs dispensed to all citizens covered by the NHS. Administrative data collection, managed at a regional level, is nationally standardized, extremely accurate, and commonly used for epidemiological research in Italy and elsewhere [23,54]. However, it should be acknowledged that these databases are not a primary source of health data, as the information is collected for administrative purposes, and they do not contain variables of potential interest, such as the patient’s clinical history, data relating to lifestyle habits, or indication for treatments. It should also be noted that drugs not reimbursed by the NHS, including some specialist drugs and all non-prescription drugs, are not tracked. Moreover, the choice of the 2012–2018 period was mainly due to the availability of administrative data. However, the COVID-19 pandemic could have modified the prescribing patterns, making challenging the assessment of inappropriate prescribing. Finally, it should be pointed out that the analysis was carried out in a single Italian region, albeit with a high resident population, and that different regional contexts could show different trends, based on local health policies.

The ERD list is based on PIPs reported in the 2015 Beers criteria, the STOPP and START criteria, and the EU-(7)-PIM list. While this approach allowed us to include a large number of known PIPs, it should be kept in mind that our knowledge on possible inappropriate uses of medications increases every day. New methods based on the evaluation of drugs (in particular psychotropics) according to their receptor affinity hold the promise of better defining inappropriate prescribing in the near future [55,56].

## 5. Conclusions

Overall, inappropriate prescribing affected one in three older persons in our primary care setting, with an even higher prevalence in females and in people aged 75–94 years.

Our systematic evaluation of PIPs over a period of 7 years showed a trend of decreasing prevalence. This is certainly a positive result, which must be monitored over time and compared with other regional and national contexts. Despite the decreasing trend both in the overall prevalence and in the most frequently reported inappropriate drugs, however, the point values and the stable rank suggest the need for policymakers to implement effective actions to face common and preventable inappropriate prescribing in primary care, promoting approaches such as medication review and making updated information and support tools available to prescribers. In this context, the evaluation of the most commonly involved specific PIPs can be useful for healthcare administrators and policymakers to better finalize corrective interventions. Moreover, the evidence of differences by sex and age classes suggests the need for personalized strategies.

Given the central role that primary care plays in coordinating healthcare, our findings highlight the need to prioritize intervention in this setting as a key strategy to reduce iatrogenic medication-related harm in the current healthcare system, especially in older people.

## Figures and Tables

**Table 1:** Characteristics of outpatients older than 65 years exposed to at least one potentially inappropriate prescription in Piedmont in the period 2012–2018.

	2012	2013	2014	2015	2016	2017	2018
Total, N	418,537	429,670	408,378	389,848	375,812	363,138	339,764
Males, N (%)	161,592 (38.61)	167,324 (38.94)	158,343 (38.77)	151,416 (38.84)	145,988 (38.85)	141,551 (38.98)	132,608 (39.03)
Age, mean (SD)	75.69 (7.17)	75.77 (7.25)	75.83 (7.29)	75.89 (7.29)	75.98 (7.32)	76.05 (7.32)	76.14 (7.34)
Age ≥80 years, N (%)	109,128 (26.07)	114,621 (26.68)	110,079 (26.96)	106,047 (27.20)	103,899 (27.65)	10,1170 (27.86)	95,930 (28.23)
Number of medications per patient, median (Q1–Q3)	6 (4–9)	6 (4–9)	6 (4–9)	6 (4–9)	6 (4–9)	6 (4–9)	6 (4–9)
≥5 medications, N (%)	295,897 (70.70)	309,414 (72.01)	295,746 (72.42)	282,162 (72.38)	270,979 (72.10)	262,066 (72.17)	242,898 (71.49)
≥10 medications, N (%)	74,229 (17.74)	79,937 (18.60)	76,581 (18.75)	72,428 (18.58)	69,058 (18.38)	66,216 (18.23)	58,790 (17.30)

**Table 2:** Characteristics of outpatients older than 65 years exposed to at least one potentially inappropriate prescription in Piedmont in the period 2012–2018.

Rank	2012	2013	2014	2015	2016	2017	2018
1.	Diclofenac	Proton Pump Inhibitors	Diclofenac	Diclofenac	Diclofenac	Diclofenac	Diclofenac
% drugs’ users	10.71%	11.31%	10.13%	9.73%	10.02%	9.93%	9.11%
%patients with PIPs	24.74%	25.90%	24.85%	25.16%	27.09%	27.90%	27.40%
2.	Proton pump inhibitors	Diclofenac	Proton Pump Inhibitors	Proton pump inhibitors	Proton pump inhibitors	Proton pump inhibitors	Proton pump inhibitors
% drugs’ users	8.80%	10.51%	8.49%	7.75%	6.53%	6.37%	5.82%
%patients with PIPs	20.34%	24.08%	20.82%	20.04%	17.66%	17.88%	17.52%
3.	Ketoprofen	Ketoprofen	Ketoprofen	Ketoprofen	Ketoprofen	Ketoprofen	Ketoprofen
% drugs’ users	5.87%	5.80%	5.44%	4.91%	4.51%	4.09%	3.61%
%patients with PIPs	13.58%	13.29%	13.35%	12.69%	12.20%	11.49%	10.85%
4.	Ticlopidine	Tramadol	Tramadol	Tramadol	Insulin	Insulin	Insulin
% drugs’ users	4.03%	3.60%	3.44%	3.15%	3.01%	2.97%	2.83%
%patients with PIPs	9.31%	8.25%	8.43%	8.15%	8.13%	8.35%	8.51%
5.	Tramadol	Ticlopidine	Ticlopidine	Insulin	Tramadol	Paroxetine	Paroxetine
% drugs’ users	3.76%	3.53%	3.07%	3.04%	2.97%	2.86%	2.81%
%patients with PIPs	8.68%	8.09%	7.54%	7.87%	8.03%	8.04%	8.45%

PIPs: potentially inappropriate prescriptions.

**Table 3:** Characteristics of outpatients older than 65 years exposed to at least one potentially inappropriate prescription in Piedmont in the year-2018.

Rank	Total	Men	Women	65–75 y	75–85 y	85–95 y	≥95 y
1.	Diclofenac	Diclofenac	Diclofenac	Diclofenac	Diclofenac	Proton pump inhibitors	Proton pump inhibitors
% drugs’ users	9.11%	7.84%	10.07%	9.41%	9.57%	7.22%	6.14%
%patients with PIPs	27.4%	25.98%	28.31%	30.23%	26.53%	21.58%	22.93%
2.	Proton pump inhibitors	Proton pump inhibitors	Proton pump inhibitors	Proton pump inhibitors	Proton pump inhibitors	Diclofenac	Diclofenac
% drugs’ users	5.82%	5.63%	5.97%	4.67%	6.78%	7.14%	4.32%
%patients with PIPs	17.52%	18.67%	16.78%	15.02%	18.81%	21.34%	16.14%
3.	Ketoprofen	Insulin	Ketoprofen	Ketoprofen	Ketoprofen	Tramadol	Ticlopidine
% drugs’ users	3.61%	3.24%	3.94%	3.94%	3.56%	3.16%	3.03%
%patients with PIPs	10.85%	10.75%	11.08%	12.65%	9.87%	9.46%	11.32%
4.	Insulin	Ketoprofen	Paroxetine	Paroxetine	Insulin	Insulin	Tramadol
% drugs’ users	2.83%	3.17%	3.66%	2.61%	3.24%	3.01%	2.38%
%patients with PIPs	8.51%	10.5%	10.30%	8.40%	8.98%	8.99%	8.87%
5.	Paroxetine	Tramadol	Tramadol	Insulin	Paroxetine	Paroxetine	Insulin
% drugs’ users	2.81%	1.84%	2.93%	2.47%	3.07%	2.84%	2.21%
%patients with PIPs	8.45%	6.11%	8.23%	7.95%	8.51%	8.48%	8.26%

PIPs: potentially inappropriate prescriptions.

**Table 4:** Odds ratio (OR) and 95% confidence intervals (95%CI) for the association between age, sex, and calendar year and the probability of being exposed to at least one PIP.

Variable	OR	95%CI
Age		
65–75 y	1	ref
75–85 y	1.144	(1.138–1.150)
85–95 y	1.083	(1.075–1.091)
≥95 y	0.886	(0.867–0.905)
Sex		
Males	1	ref.
Females	1.245	(1.237–1.253)
Year	0.944	(0.943–0.945)

## Data Availability

The pooled data that support the findings of this study are available from the corresponding author, M.C., upon reasonable request.

## Appendix A

**Table A1:** Full list of potentially inappropriate prescriptions (PIP) considered in the study.

**ATC**	**Drug**	**PIP**
A02BC01	Omeprazole (PPI > 8 weeks)	Proton pump inhibitor
A02BC02	Pantoprazole (PPI > 8 weeks)	Proton pump inhibitor
A02BC03	Lansoprazole (PPI > 8 weeks)	Proton pump inhibitor
A02BC04	Rabeprazole (PPI > 8 weeks)	Proton pump inhibitor
A02BC05	Esomeprazole (PPI > 8 weeks)	Proton pump inhibitor
A10AB01	Insulin (human)	Insulin
A10AB04	Insulin, lispro	Insulin
A10AB05	Insulin, aspart	Insulin
A10AB06	Insulin, glulisine	Insulin
A10AE01	Insulin (human)	Insulin
A10AE02	Insulin (beef)	Insulin
A10AE03	Insulin (pork)	Insulin
A10AE04	Insulin glargine	Insulin
A10AE05	Insulin detemir	Insulin
A10AE06	Insulin degludec	Insulin
A10AE30	Insulin combinations	Insulin
A10AE54	Insulin glargine and lixisenatide	Insulin
A10AE56	Insulin degludec and liraglutide	Insulin
A10BB01	Glibenclamide	Glibenclamide
A10BB07	Glipizide	Glipizide
A10BB12	Glimepiride	Glimepiride
A10BD02	Glibenclamide combination	Glibenclamide
A10BD05	Pioglitazone and metformin	Pioglitazone
A10BD06	Glimepiride and Pioglitazone	Pioglitazone
A10BD06	Glimepiride and Pioglitazone	Glimepiride
A10BD09	Pioglitazone and alogliptin	Pioglitazone
A10BF01	Acarbose	Acarbose
A10BG03	Pioglitazone	Pioglitazone
B01AA07	Acenocoumarol	Acenocoumarol
B01AC05	Ticlopidine	Ticlopidine
B01AC56	Acetylsalicylic acid, combinations with proton pump inhibitors	Proton pump inhibitors
C01AA08	Metildigoxin	Metildigoxin
C01BA03	Disopyramide	Disopyramide
C01BC03	Propafenone	Propafenone
C01BC04	Flecainide	Flecainide
C02AB01	Methyldopa	Methyldopa
C02AC05	Moxonidine	Moxonidine
C08CA05	Nifedipine	Nifedipine
G02CB03	Cabergoline	Cabergoline
G03AA09	Desogestrel and ethinylestradiol	Ethinylestradiol
G03AA10	Gestodene and ethinylestradiol	Ethinylestradiol
G03AB06	Gestodene and ethinylestradiol	Ethinylestradiol
G03BA03	Testosterone	Testosterone
G03CA01	Ethinylestradiol	Ethinylestradiol
G03CA03	Estradiol	Estradiol
G03CA04	Estriol	Estriol
G03CA09	Promestriene	Promestriene
G03CX01	Tibolone	Tibolone
G03FA01	Norethisterone and estrogen	Estradiol
G03FA11	Levonorgestrel and estrogen	Estradiol
G03FA14	Dydrogesterone and estrogen	Estradiol
G03FA17	Drospirenone and estrogen	Estradiol
G03FB05	Norethisterone and estrogen	Estradiol
G03FB08	Dydrogesterone and estrogen	Estradiol
G03FB09	Levonorgestrel and estrogen	Estradiol
G03FB12	Nomegestrol and estrogen	Estradiol
H01BA02	Desmopressin	Desmopressin
L02AB01	Megestrol	Megestrol
M01AB01	Indometacin	Indometacin
M01AB05	Diclofenac	Diclofenac
M01AB15	Ketorolac	Ketorolac
M01AB16	Aceclofenac	Aceclofenac
M01AC01	Piroxicam	Piroxicam
M01AC05	Lornoxicam	Lornoxicam
M01AC06	Meloxicam	Meloxicam
M01AE03	Ketoprofen	Ketoprofen
M01AE09	Flurbiprofen	Flurbiprofen
M01AX01	Nabumetone	Nabumetone
N02AD01	Pentazocine	Pentazocine
N02AX02	Tramadol	Tramadol
N03AA02	Phenobarbital	Phenobarbital
N03AB02	Phenytoin	Phenytoin
N03AE01	Clonazepam	Clonazepam
N03AX11	Topiramate	Topiramate
N04AA01	Trihexyphenidyl	Trihexyphenidyl
N04AA02	Biperiden	Biperiden
N04AB02	Orphenadrine	Orphenadrine
N04BC01	Bromocriptine	Bromocriptine
N05AC01	Propericiazine	Propericiazine
N06AA02	Imipramine	Imipramine
N06AA04	Clomipramine	Clomipramine
N06AA06	Trimipramine	Trimipramine
N06AA09	Amitriptyline	Amitriptyline
N06AA10	Nortriptyline	Nortriptyline
N06AB03	Fluoxetine	Fluoxetine
N06AB05	Paroxetine	Paroxetine
N06AB08	Fluvoxamine	Fluvoxamine
N06BA04	Methylphenidat	Methylphenidat
R06AD02	Promethazine	Promethazine
